# The causes of pulmonary hypertension and the benefits of aerobic exercise for pulmonary hypertension from an integrated perspective

**DOI:** 10.3389/fphys.2024.1461519

**Published:** 2024-10-17

**Authors:** Yinping Song, Hao Jia, Qing Ma, Lulu Zhang, Xiangyi Lai, Youhua Wang

**Affiliations:** ^1^ School of Physical Education, Xi’an Fanyi University, Xi’an, China; ^2^ School of Physical Education, Shaanxi Normal University, Xi’an, China

**Keywords:** pulmonary hypertension, aerobic exercise, ER stress, ROS, thrombosis, 6-min walk distance

## Abstract

Pulmonary hypertension is a progressive disease of the pulmonary arteries that begins with increased pulmonary artery pressure, driven by progressive remodeling of the small pulmonary arteries, and ultimately leads to right heart failure and death. Vascular remodeling is the main pathological feature of pulmonary hypertension, but treatments for pulmonary hypertension are lacking. Determining the process of vascular proliferation and dysfunction may be a way to decipher the pathogenesis of pulmonary hypertension. In this review, we summarize the important pathways of pulmonary hypertension pathogenesis. We show how these processes are integrated and emphasize the benign role of aerobic exercise, which, as an adjunctive therapy, may be able to modify vascular remodeling in pulmonary hypertension.

## Introduction

Pulmonary hypertension (PH) is a severe pulmonary vascular disease characterized by excessive vascular cell proliferation, increased extracellular matrix deposition, and accumulation of inflammatory cells within the walls of the pulmonary vasculature, which contribute to increased pulmonary vascular resistance (Pullamsetti et al., 2017). During the pathologic progression of PH, there is a progressive narrowing of the pulmonary lumen and an increase in pulmonary artery pressure, which ultimately leads to right ventricular failure and death (Humbert et al., 2019). The Seventh World Symposium on Pulmonary Hypertension has held in Barcelona. It is endorsed by several patients’ organizations, the European Respiratory Society, the International Society for Heart and Lung Transplantation, the Société de Pneumologie de Langue Française and the European Reference Network for Rare Lung Diseases (ERN-LUNG). In recent years, the European Commission has launched programmes for a higher level of research and care in the field of rare diseases. Symbolically, the 2024 Symposium started for the first time with the patients’ perspective, showing the respect and importance given to patients’ priorities. The translational aspects of modern pulmonary vascular research have been highlighted by the development of novel treatment approaches directly targeting the basic drivers of pulmonary vascular remodeling through activin signaling inhibition (Humbert et al., 2023). Similarly, refined treatment approaches for PH highlighted the complementarity of the different treatment modalities targeting different mechanisms of the disease.

In previous studies, PH is classified according to the level of pulmonary artery pressure; it was considered mild to moderate when the pulmonary artery pressure is between 25 and 45 mmHg, and severe PH when it exceeds 45–50 mmHg. The Sixth World Symposium on PH recommended that the diagnosis be made by setting the minimum mean pulmonary artery pressure from 25 mmHg to 20 mmHg (Simonneau et al., 2019). Growing evidence that even a mean pulmonary artery pressure slightly above this threshold is associated with an increased risk of death (Maron et al., 2016; Assad et al., 2017; Douschan et al., 2018), irrespective of the underlying causative factor. This provides more opportunities and options for treating patients in the early stages of the disease.

The previous of clinical treatments for PH remain vasodilators (Goldenberg et al., 2019). Currently, drug development for pulmonary hypertension is undergoing a dramatic shift, with the advent of novel drugs such as tyrosine kinase inhibitors and activin receptor ligand traps (including sotatercept, which was approved by the FDA in March 2024 for the treatment of PH in adults). Sotatercept is a fusion protein that traps activins and growth differentiation factors involved in pulmonary arterial hypertension. In patients with pulmonary arterial hypertension who were receiving stable background therapy, sotatercept resulted in a greater improvement in exercise capacity as assessed by the 6-minute walk test (Hoeper et al., 2023). But, sotatercept-like drugs might be detrimental effects in the heart.

The 3-year survival rate for patients with PH is 68%–70% (Hurdman et al., 2012; Farber et al., 2015). Current therapies mainly target pulmonary vasoconstriction and do not effectively intervene or reverse pulmonary arterial vascular remodeling. Therefore, there is an urgent need for new explorations to directly address targeting pulmonary artery remodeling in this PH pathology.

In addition to the extensive research on medication, little is known about the impact of lifestyle on PH. Currently, complex exercise rehabilitation interventions are being developed for patients with pulmonary hypertension: the Supervised Pulmonary Hypertension Exercise Rehabilitation (SPHERe) trial is also underway (Ennis et al., 2023). Notably, aerobic exercise is effective against skeletal and cardiac dysfunction in PH (Vieira et al., 2020). Exercise rehabilitation training as an adjunct to disease-specific treatments is often effective and safe for patients with PH (Dong and Li, 2022). It can improve exercise capacity and endurance, skeletal and respiratory muscle performance, cardiorespiratory function and quality of life in patients with PH. Therefore, the aim of this review is to investigate the pathways at the level of pathomechanisms and to illustrate the efficacy and safety of performing aerobic exercise in order to provide a theoretical basis for the prevention and treatment of PH.

## Pulmonary hypertension and pulmonary artery remodeling

Vascular dysfunction and vascular remodeling are thought to be central mechanisms in the development of high pulmonary arterial pressure, which is chronically elevated by dysregulation of functional vascular signaling pathways driven by relevant triggers. This process leads to high afterload and failure of the right ventricle. The process of pulmonary vascular remodeling involves all the layers of the membrane structure of the vascular wall; the intima consists of endothelial cells, the middle layer consists mainly of smooth muscle cells (SMCs), and the tunica consists of fibroblasts. It is complicated by the presence of cellular heterogeneity within the compartments of the pulmonary artery wall.

### Pulmonary artery endothelium

Most studies have concluded that the pulmonary artery endothelium is the initial site of PH disease (Stenmark et al., 2012; Kuebler et al., 2017). Pulmonary vasoconstriction occurs early in PH pathology, in turn, vasoconstriction is closely related to endothelial disfunction. The extent to which the endothelium is compromised in PH is not fully understood. In one study, based on cross-sectional analysis of the pulmonary arteries, the normal intima accounted for approximately 10% of the total thickness. Idiopathic PH, which is typical of severe PH, shows marked thickening and disorganization of the intima; in severe PH disease, the intima of the pulmonary arteries in PH shows an increase in thickness of about three times compared to normal pulmonary arteries (Stacher et al., 2012). The thickened intima leads to increased pulmonary vascular resistance. Using the volume density parameter of alveolar septa as a reference, PH is associated with a doubling of the intima compared to more normal pulmonary arteries (Stacher et al., 2012). The types of intimal thickening are diverse. They can be briefly categorized according to the predominance of collagen and mucin, fibroblasts or endothelial cells. Endothelial-like cells proliferate abnormally in a disorganized and chaotic manner and are prone to develop lesions. Studies have shown that high expression of angiogenic markers including vascular endothelial growth factor (VEGF), VEGF receptor and hypoxia inducible factor (HIF) are detected in these diseased endothelial tissues (Bryant et al., 2012).

Hypoxia is also a major causative agent of PH, and hypoxia leads to endothelial cell dysfunction through activation of receptor-γ cofactor-1α by endothelial peroxisome proliferators, increased formation of reactive oxygen species (ROS), mitochondrial dysfunction, nuclear factor κB (NF-κB) activation, and subsequent secretion of interleukin-6 (IL-6) and tumor necrosis factor-α (TNF-α) (Ye et al., 2016). Endothelial dysfunction leads to decreased production of vasodilators such as nitric oxide (NO) and prostacyclin and increased production of vasoconstrictors such as endothelin-1 (ET-1). ET-1 is a potent vasoconstrictor, and its prolonged overexpression not only affects vascular tone but also induces vascular remodeling. Therefore, it plays an important role in the pathogenesis of PH. In addition, ET-1 is involved in lung microvascular remodeling (Stam et al., 2018). There is substantial evidence that the endothelium may play a key role as an inflammatory cell signaling hub (Pugliese et al., 2015). As a signaling hub, the endothelium maintains feed-forward interactions between resident fibroblasts and macrophages. In addition, prolonged hypoxia disabled the FIS1 deSUMOylation by diminishing the availability of SENP1 in mitochondria via inducing miR (microRNA)-138 and consequently resulted in mitochondrial dysfunction and metabolic reprogramming in pulmonary endothelium (Zhou et al., 2023). The specific mechanism(s) of the initiating stimulus(s) or injury(s) leading to abnormal endothelial cell proliferation are not fully understood, but may include hypoxia, shear stress, inflammation, or response to drugs or toxins in the context of genetic susceptibility.

### Pulmonary artery mesentery

PH is a progressive disease that is typically characterized by an abnormal proliferation of SMCs in the pulmonary artery mesentery, thus leading to progressive apoptosis of the pulmonary arteries. Numerous studies have shown that hypoxia causes pulmonary vasoconstriction and vascular remodeling (Wilkins et al., 2015; Huang et al., 2022). Prolonged exposure to hypoxia leads to hypoxic PH. Due to the superimposed release of hypoxic and growth factors, the pulmonary arteries undergo an excessive muscularization process, with mesenchymal smooth muscle cell remodeling as the main remodeling. Pulmonary artery smooth muscle cells (PASMCs) are the predominant cell type in the inner layer of the pulmonary arteries and contain contractile proteins that are regulated by calcium and control vascular tone (Lyle et al., 2017). When dysregulated, SMCs contract abnormally, which in turn leads to sustained vasoconstriction, ultimately triggering vascular smooth muscle remodeling, a disease hallmark of PH.

PASMCs are a cell type without terminal differentiation and can maintain significant plasticity (Gomez and Owens, 2012). Under hypoxia, overproliferation and anti-apoptosis of PASMCs in small pulmonary arteries can cause pulmonary artery remodeling to occur. SMCs have been shown to transmit inflammatory signals in lung tissue through their secretion of pro-inflammatory cytokines (Dreymueller et al., 2014). In addition, the proliferative and quasi-synthetic phenotypic switch observed in PASMCs under hypoxia is mediated by hypoxia-inducible factor-1(HIF-1) driven expression of miR-9-1 and miR-9-3 (Shan et al., 2014). HIF-2α is a homologue of HIF-1α, chronic hypoxia enhances HIF-2α stability, which causes increased arginase expression and dysregulates normal vascular NO homeostasis, suggesting that HIF-2α contributes to the development of hypoxic pulmonary vascular remodeling by upregulating these vasoconstrictors through the endothelium. HIF-1-dependent upregulation of miR-210 leads to anti-apoptosis in PASMCs by targeting the transcription factor E2F3 (Gou et al., 2012). Hypoxia-induced muscularization of non-muscularized pulmonary artery vessels involves pre-existing smooth muscle cell progenitors that undergo dedifferentiation, migration to distal vessels, proliferation and redifferentiation (Sheikh et al., 2014). Thus, multiple mechanisms act synergistically and ultimately contribute to the pro-proliferative, pro-growth, and anti-apoptotic phenotypes of PASMCs during the pathologic process of PH.

### Pulmonary artery epicardium

As hypoxia activates ROS signaling, it stimulates the production of α-smooth muscle actin (α-SMA), a classical marker of activated fibroblasts (Barman et al., 2014). The work of Chai et al. demonstrated that hypoxia induces the proliferation of pulmonary artery adventitia fibroblast (PAAF), migration and vascular remodeling, as observed in the pulmonary artery wall of hypoxic rats *in vivo*, where hypoxia induced medial and lateral thickening as well as excessive fibrin and collagen deposition (Chai et al., 2018).

Fibroblasts provide mechanical strength to tissues by producing extracellular matrix and providing matrix support. In addition, fibroblasts are able to respond to various stimuli such as vasodilation or hypoxia (Stenmark et al., 2013). In response to these stimuli, pulmonary artery endothelial fibroblasts exhibit a distinct pro-inflammatory phenotype characterized by an increase in chemokines, cytokines and adhesion molecules (Kitamura et al., 2011). In addition, interactions between fibroblasts and leukocytes at sites of chronic inflammation appear to promote the continued survival and retention of leukocytes, which in turn leads to a delay or disappearance of the inflammatory lesion (Buckley, 2011).

Thus, pulmonary artery endothelial cells (PAECs), PASMCs, and pulmonary artery adventitia fibroblasts (PAAFs) are able to interact cooperatively with each other in response to stimuli induced during the pathologic process of PH, such as altered fluid shear stress, stretch, and hypoxia. Pulmonary artery constriction, regulated by contraction and relaxation of PASMCs, can be modulated by paracrine factors released by PAECs. ECs release of the endothelium-derived constricting factors (EDCF), ET-1, from PAEC causes pulmonary vasoconstriction by activating ET receptors (e.g., ETA) and thromboxane/prostaglandin endoperoxide (TP) receptors, respectively, on the membrane of PASMC. Release of the endothelium-derived relaxing factors (EDRF), NO and prostacyclin (PGI2), and the endothelium-derived hyperpolarizing factors (EDHF) from PAEC (Makino et al., 2011). Serotonin synthesized by PAECs is transferred through these gap junctions to PASMCs where it activates TGF-β1 signaling, which in turn induces a more differentiated phenotype. Since TGF-β1 is an important regulator of fibrosis, this is an important way in which PAECs and PASMCs respond to PH (Gairhe et al., 2012).

## Causes and mechanisms of pulmonary hypertension

### Pulmonary hypertension and hypoxia

In the pathological progression of PH, chronic hypoxia, vasoconstriction, endothelial dysfunction, mitochondrial abnormalities, and inflammation are among the many factors that can activate the HIF signaling pathway, which in turn triggers alterations in the intima-media, endothelium, and epima-media of the pulmonary vasculature, inflammatory cells, and cardiomyocytes, leading ultimately to pulmonary vascular remodeling and right ventricular failure.

Hypoxic environments are widely used as a major stimulus for proliferative vasculopathy in PH models prepared in small animals; hypoxic environments also trigger reversible pulmonary vascular remodeling when humans arrive to reach high altitudes (Sydykov et al., 2021). Cellular sensing of oxygen is multilayered and tissue-specific, but the hypoxia-inducible transcription factors HIF-1α and HIF-2α are key regulators of hypoxic adaptation in pulmonary vascular cells (Semenza, 2012).

It has been shown that HIF-1α expression is upregulated in the pulmonary arteries of patients with PH (Labrousse-Arias et al., 2015). The cellular sources of increased HIF-1α expression in lung tissues of patients with PH are pulmonary artery endothelial cells (Bryant et al., 2016) and pulmonary artery SMCs (Kurosawa et al., 2019; Marsboom et al., 2012). The proliferation and quasi-synthetic phenotypic switch observed in PASMCs under hypoxia is mediated by HIF-1 drive (Shan et al., 2014).

### Pulmonary hypertension and inflammation

Clinical results have shown that the degree of perivascular inflammation in PH patients correlates with pulmonary hemodynamics and vascular remodeling (Stacher et al., 2012). In lung biopsies from PH patients, almost all inflammatory cell lineages were found in the vicinity of remodeled pulmonary vessels, such as macrophages, mast cells, T lymphocytes, B lymphocytes, dendritic cells, and neutrophils (Savai et al., 2012).

Inflammatory mechanisms play an important role in PH pathology. Autoantibodies (e.g., antinuclear antibodies), and elevated levels of pro-inflammatory cytokines Interleukin-1 (IL-1) and IL-6 were detected in some patients with PH. Histologic findings in the lungs have also shown an inflammatory infiltrate (macrophages and lymphocytes) at the lesion in patients with severe PH (Yaku et al., 2022). Interleukin-1 β (IL-1β) is a key inflammatory cytokine released in response to inflammasome activation and is an important mediator of the inflammatory response. Elevated serum levels of IL-1β in patients with PH are associated with worsening PH (Pickrell and Youle, 2015). IL-1β may be derived in part from neutrophils and T cells infiltrated in diseased pulmonary vessels, a key component of chronic hypoxia-induced inflammation in PH mice, i.e., it contains Pyrin domain 3 (NLRP3) of the Nod-like receptor family and contains the positive staining for apoptosis-associated speckled protein (ASC) of the caspase recruitment domain proved this (Cero et al., 2015). Elevations in lung IL-1β, IL-1βR and MyD88 preceded pulmonary hypertension in hypoxic mice. Knockdown of IL-1βR or a molecular adaptor of mouse myeloid differentiation primary response protein 88 prevented hypoxia-induced PH (Parpaleix et al., 2016). In PASMCs, prostacyclin modulates vasodilatation and has antiproliferative effects. This vasodilatory effect is mediated through the second messenger cyclic adenosine monophosphate (cAMP). IL-1β attenuates the conversion of ATP to cAMP in PASMCs by down-regulating adenylate cyclase. In addition, IL-1β can regulate PASMC growth through the IL-1R1/MyD88 pathway (Yang et al., 2014).

As can be seen, inflammatory factors are clearly drivers and contributors to the pathologic process of PH. some of these inflammatory factors show a strong correlation with the severity of PH disease (notably interleukins IL-1, IL-6 (Hu et al., 2020). Not only is inflammation part of the pathology of PH, but inflammation may indeed drive several key pulmonary vasculopathic features of the disease.

### PH and ROS

ROS, including O_2-_ and H_2_O_2_, play important roles as mediators and signaling molecules capable of activating multiple pathways involved in the control of pulmonary vascular tone, cell proliferation and apoptosis, inflammation, and fibrosis. Supraphysiologic doses of H_2_O_2_, in turn, are highly correlated with pathophysiologic responses leading to vasoconstriction and PH.

In the pulmonary vasculature, Nox isozymes can be activated by a number of stimuli (e.g., G-protein-coupled receptor agonists, angiotensin II, thrombin, endothelin, 5-hydroxytryptophan, thromboxane A2 (Karpińska et al., 2017). Nox-derived ROS are able to activate NF-κβ, activate MAPK, and cause aberrant cell proliferation, as well as potassium channel regulation in response to changes in oxygen concentration (Dias-Freitas et al., 2016; Brendel et al., 2020). Both Nox1 and Nox4 induce proliferation in PASMCs (Mittal et al., 2007). Nox4 expression in PASMCs can be induced by several stimuli, including hypoxia, shear stress, and endoplasmic reticulum stress (Bedard and Krause, 2007; Lambeth et al., 2007). The effect of Nox4 on the expression of smooth muscle differentiation markers is required, and there is a correlation between the reduction of Nox4 expression and the loss of smooth muscle differentiation markers, such as smooth α-actin and myosin heavy chain, over multiple passages in PASMCs (Clempus et al., 2007; Sturrock et al., 2006), a process in which Nox4 is involved through the induction of TGF-β1 (Sturrock et al., 2006). In PASMCs TGF-β activation of Nox4 leads to intracellular ROS production (Waghray et al., 2005). Under hypoxic conditions Nox4 increases ROS production, stimulates proliferation of PASMCs and inhibits apoptosis in lung fibroblasts (Li et al., 2008).

### Pulmonary hypertension and endoplasmic reticulum stress

Endoplasmic reticulum (ER) stress has been a hot topic in the study of disease mechanisms. Research surrounding ER stress and PH has consequently been initiated, with numerous findings suggesting that the unfolded protein response (UPR) plays an important role in the onset and progression of PH pathology. Nowadays, inhibition of ER stress is considered as a new potential intervention in the clinical treatment of PH. Several studies have demonstrated that treatments that reduce ER stress through the use of chemical chaperones can reverse or treat PH in animal models (Wu et al., 2016; Dromparis et al., 2013; Martin and Pabelick, 2014).

It has been shown that vascular remodeling is closely related to ER stress, especially to the abnormal proliferation of PASMCs (Koyama et al., 2014). It has been suggested that proliferation of PASMCs and resistance to apoptosis are critical for vascular remodeling in PH (Masson et al., 2022; Perros et al., 2008; Wang et al., 2014). ER stress is a fundamental cellular response that promotes the proliferation of PASMCs and enhances inflammatory responses (Chen et al., 2018). Therefore, it has been suggested that abnormal proliferation of PASMCs is the most important cause of pulmonary vascular remodeling in PH (Santos-Ribeiro et al., 2016; Yu et al., 2017). Studies have shown that autophagy is involved in the pathogenesis and development of MCT-induced PH (Long et al., 2013; Feng et al., 2021). And eIF2α can activate ER autophagy after ER stress. Meanwhile, eIF2α plays a key role in regulating cell proliferation and hypertrophy, and is involved in regulating the proliferation and migration of vascular smooth muscle (Jiang et al., 2016; Liu et al., 2013). All of the above suggest that ER stress is involved in the abnormal proliferation of PASMCs in PH. Inflammatory cell recruitment and persistence of inflammation in PH are two key components of pathological vascular remodeling (Hassoun et al., 2009). Activation of UPR and production of pro-inflammatory biomolecules were due to ET-1 signaling in rat PASMCs (Yeager et al., 2012). The occurrence of ER stress promotes the proliferation and inflammatory state of PASMCs, which contributes to PH pathology. The persistent inflammation produced by PASMCs is associated with vascular remodeling, and inhibiting this process may be a potential approach for treating PH. Although the function of ER stress in PASMCs has been extensively studied, the mechanism remains incompletely understood. In conclusion, inhibition of the aforementioned proliferation of pulmonary artery smooth muscle cells due to ER stress is also a potential therapeutic option for PH.

### Pulmonary hypertension and platelet activation, thrombosis

Patients with PH may even develop thrombotic arteriopathy, which indicates a prolonged activation of platelets and endothelial cells, with the continuous involvement of coagulation factors. This endothelial activation is associated with endothelial cell proliferation and plexiform lesion formation (Guignabert and Dorfmüller, 2017).

Patients with PH tend to exhibit thrombocytopenia, and those with moderate to severe disease have a poorer prognosis, suggesting that platelets appear to play a role in disease progression. Impaired platelet function in patients with PH leading to their chronic activation and degranulation, increased expression of thrombopoietin in the pulmonary arteries, and an increase in the mean platelet volume suggest that platelet activation, aggregation, and depletion may be increased in the pulmonary circulation (Vrigkou et al., 2019; Zheng et al., 2015). Platelet depletion also stimulates platelet production in the bone marrow, resulting in more platelets and potentially stronger thrombogenic effects. Activated endothelial cells may promote thrombosis through von Willebrand factor (vWF)-mediated platelet activation, factor X activation and tissue factor (TF) production (Nogueira-Ferreira et al., 2014). In rodent models of PH, the activity of this protein is positively correlated with pathological hemodynamic changes and vascular remodeling. The pathological role of TF is not only related to its prothrombotic effects, but also to its direct proliferative and migratory effects. In addition, decreased histone trimethylation and increased histone acetylation of the vWF promoter in the PH endothelium facilitated the binding of NF-κB2 to the vWF promoter and drove the transcription of vWF. epigenetic regulation of the vWF promoter contributes to the creation of a localized environment that is conducive to *in situ* thrombosis in pulmonary arteries. It reveals a direct link between inflammatory pathways and platelet adhesion in the pulmonary vascular wall, suggesting a possible role of *in situ* thrombosis in the development or progression of PH (Manz et al., 2022).

Microparticles derived from platelets and the endothelium are an increasingly well-recognized signal in PH (Zheng et al., 2015). The activation of platelets, apart from releasing granules, increases the surface expression of various adhesion molecules and receptor (e.g., selectin P, gp IIIa/IIb) as well as the production of thromboxane A2 (TxA2), which in turn activates other platelets and promotes vasoconstriction and local thrombosis (Kazimierczyk and Kamiński, 2018). Evidence of platelet function abnormalities and dysregulation of the coagulation cascade have been found in PH patients (Bazan and Fares, 2018). Coagulation processes are involved in most of the major pathophysiological pathways of PH, either directly (e.g., thrombus formation and thrombotic arteriopathy) or indirectly (through the production and release of vasoactive substances) (Kazimierczyk and Kamiński, 2018). Therefore, platelets are activated and function abnormally, platelet aggregation is diminished, there is catabolism, there are defects in the initiation of the coagulation process and propagation of clots, and there is diminished thrombin formation in patients with PH. The procoagulant activity is impaired, which may be due to the sustained and prolonged activation of the procoagulant process (Vrigkou et al., 2020). PH patients have higher levels of procoagulant microparticles (MPs) in the pulmonary vasculature, which are fragments of circulating cell membranes released by activated and/or apoptotic cells that stimulate thrombosis by providing coagulation factor activation (Nadaud et al., 2013). As a result, patients with PH have reduced levels of PGI2 and increased levels of TxA2. Upon activation, platelets release TxA2 and platelet-derived growth factor (PDGF), among others, which induce activation of nearby platelets and contribute to platelet amplification and aggregation. Meanwhile, PGI2, which is mainly produced by inactivated endothelium, has vasodilatory and antiplatelet aggregation effects. Thus, this imbalance appears to be the result of impaired endothelial function and/or enhanced platelet activation in the pulmonary vasculature, with important implications for disease progression.

### Pulmonary hypertension and genetics

Considerable progress has been made in the genomics of pulmonary arterial hypertension since the 6th World Symposium on Pulmonary Hypertension, with the identification of 17 genes, as well as common variants that confer a modest increase in PH risk. Gene and variant curation by an expert panel now provides a robust framework for knowing which genes to test and how to interpret variants in clinical practice. Researchers recommend that genetic testing be offered to specific subgroups of symptomatic patients with PH, and to children with certain types of group 3 PH. Testing of asymptomatic family members and the use of genetics in reproductive decision-making require the involvement of genetics experts (Austin et al., 2024). In 2020, investigators for the United States Pulmonary Hypertension Scientific Registry (USPHSR) provided data from the first US PH patient registry to include genetic information. Genetic testing identified pathogenic or suspected pathogenic variants in 67 of 499 (13%) USPHSR participants (Badlam et al., 2021).

Pathogenic mutations in BMPR2 are the most common cause of heritable pulmonary arterial hypertension (HPAH) in both adults and children (Welch and Chung, 2020). The loss of BMPR2 promotes endothelial dysfunction, endothelial to mesenchymal transition and pulmonary arterial smooth muscle cell hyperproliferation (Theilmann et al., 2020; Hiepen et al., 2019; Hopper et al., 2016). Genes with causal mutations identified in children with PH differs from the prevalence of causal gene mutations identified in adults with PH. For example, mutations in TBX4, a transcription factor in the T-box gene family that modulates lung development, are found more commonly in children with PH (Zhu et al., 2018).

Clinical observations, molecular discoveries, and laboratory technology have made genetic counseling and testing possible for patients diagnosed with PH, especially group 1 PAH. Recent studies in scRNA-seq have significantly deepened our understanding of PH by revealing cellular heterogeneity and uncovering key molecular pathways involved in disease progression. Dysregulated endothelial cell subpopulations with proliferative and angi ogenic phenotypes contribute to aberrant vascular remodeling, as exemplified by the role of CD74 in endothelial dysfunction—its knockdown regulates endothelial cell proliferation and barrier integrity, suggesting a potential therapeutic target (Rodor et al., 2022). Additionally, scRNA-seq analyses have identified significant changes in immune cells: macrophages interact with other disease-related cells via the PI3K/Akt pathway (Miao et al., 2022); T cells and natural killer cells drive heightened inflammation through upregulation of CCL5 (Kumar et al., 2024); and neutrophil subsets expressing high levels of MMP9 correlate with increased mortality in idiopathic pulmonary arterial hypertension patients (Zhang et al., 2023). These insights highlight the complex interplay between endothelial dysfunction and immune-mediated inflammation in PH, offering new potential therapeutic targets, particularly in immune cell regulation and endothelial function. Furthermore, to advance gene- and pathway-specific care and targeted therapies, gene-specific registries will be essential to support patients and their families and to lay the foundation for genetically informed clinical trials.

**FIGURE 1:**
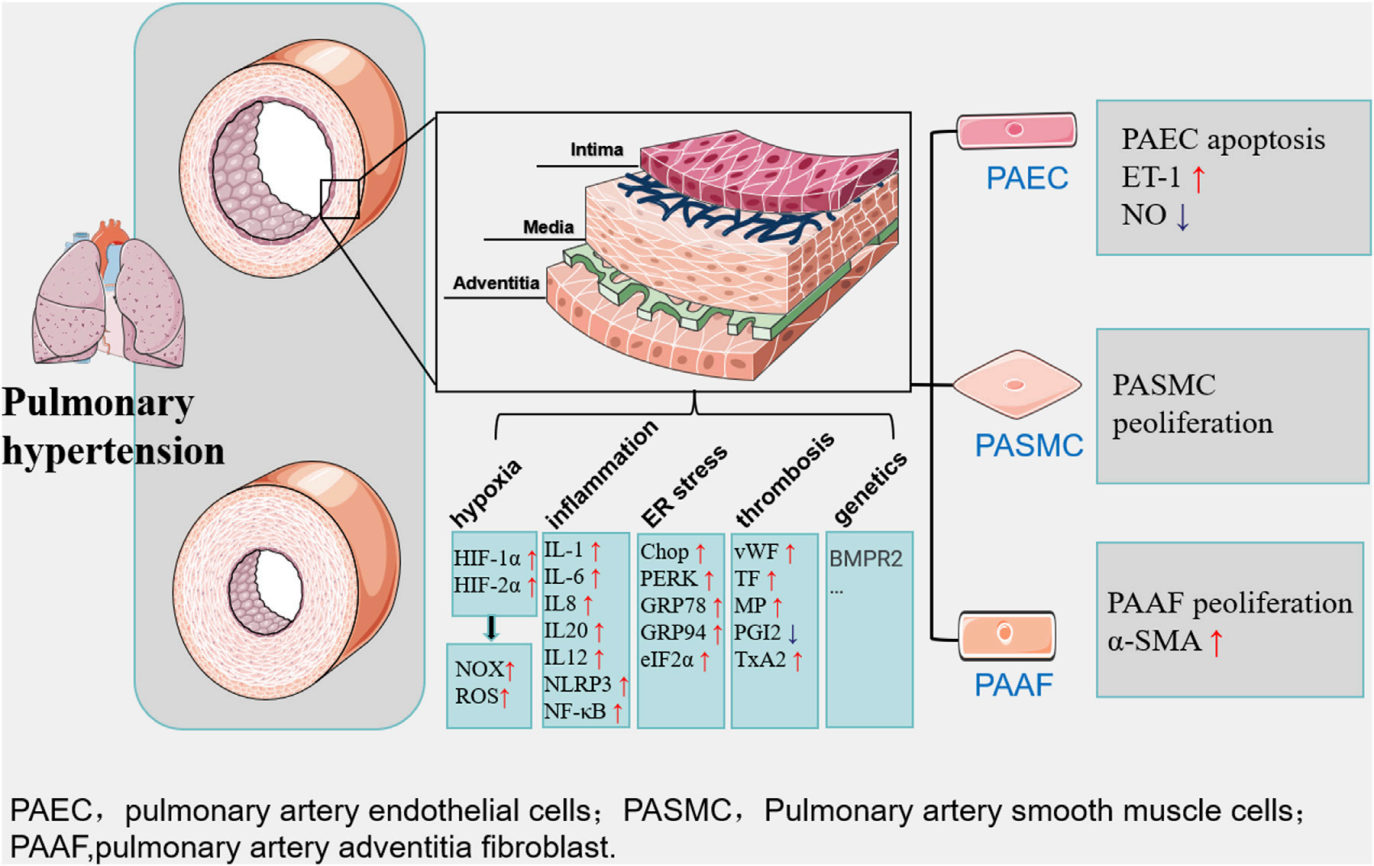
Multiple factors such as hypoxia, inflammation and ER stress lead to long-term maladaptation and dysfunction of the pulmonary artery vasculature, ultimately leading to the development of pulmonary hypertension. The main manifestations are changes in the structure and function of vascular endothelial cells, smooth muscle cells, and extracellular matrix.

In conclusion, the causes of pulmonary arterial hypertension include increased vascular resistance, etc., of which hypoxia is the most important factor in the formation of pulmonary arterial hypertension, under the effect of hypoxia, inflammation, ROS, ER stress, platelet activation, thrombosis and genetics together lead to pathological changes in pulmonary arterial vascular configuration (Figure 1).

## Aerobic exercise and pulmonary hypertension

Aerobic exercise has emerged as adjuvant therapy for many diseases, and the beneficial relationship between aerobic exercise and lung disease has gradually become closer.

### Benefits of aerobic exercise to pulmonary vascular membrane

Based on the existing literature, it seems that researchers prefer to discuss the benefits of aerobic exercise on each layer of the vessel separately rather than as a whole, and these beneficial effects are mainly concentrated in the pulmonary artery intima and pulmonary artery media.

Aerobic exercise training opposes endothelial dysfunction via enhanced endothelial NO synthase and increased NO production and bioactivity, which improves NO-dependent vasodilation of large conduit vessels. As such, exercise training improves vascular endothelial function (Fiuza-Luces et al., 2018). Exercise training studies also demonstrate that exercise training is associated with an increase in endothelial function (Ramos et al., 2015). Long-term aerobic exercise training also induces shear stress-mediated arterial remodeling that results in larger conduit and peripheral artery sizes. Aerobic exercise also reduces the wall thickness of conduit arteries (Hellsten and Nyberg, 2015). These vascular structural adaptations markedly increase the luminal reserve of the vessel and reduce the probability of a flow-limiting stenosis (Fiuza-Luces et al., 2018).

### Benefits of aerobic exercise to mechanisms of disease

Aerobic exercise and inflammation: Aerobic exercise can facilitate lung regeneration with mild inflammatory effect, and proper aerobic exercise is beneficial for lung damage repair and regeneration, which should be considered as an ideal supportive therapy for patients with different respiratory diseases (Wu et al., 2022).

Aerobic exercise and ROS: Exercise improves endothelial function and arterial stiffness by reducing inflammatory and oxidative damage signaling in vascular tissue together with an increase in antioxidant enzymes and nitric oxide availability, globally promoting functional performance (El et al., 2022). In the case of drug resistance, effective aerobic exercise could reduce ROS, activate SOD, inhibit HIF-1 and acetaldehyde dehydrogenase 1 (ALDH1), and cause a reduction in cancer stem cells to sensitise cells to drug again and ultimately inhibit the malignant proliferation of tumours. Therefore, in the treatment of lung adenocarcinoma, the inhibitory effect of aerobic exercise on oxidative stress can be used as an effective adjunct measure in the treatment of lung adenocarcinoma (Yang et al., 2022).

Aerobic exercise and ER stress: Study showed 8-week exercise and choline intervention also inhibited the protein expression of myocardial MFN2, PERK/eIF2α/ATF4, and NLRP3/caspase-1/IL-1β signaling pathways, thereby effectively reducing mitochondrial fusion, endoplasmic reticulum stress, and inflammation. Aerobic exercise increases can improve cardiac function in cardiovascular disease (CVD) rats (Ma et al., 2021).

Aerobic exercise and thrombosis: Exercise is considered a double-edged sword since, on one hand, acute exercise can be a direct cause of a thrombotic event, and on the other hand, exercise training is a potent intervention for lowering the risk of cardiovascular events. Thus, for patients at risk, the safer recommendation would accordingly be to initiate exercise programs at low to moderate intensities. Studies combining platelet reactivity assay, clinical measures of hemostatic markers, and the novel functional measure of clot microstructure will provide a new level of detailed prediction of the susceptibility to harmful arterial blood clots (Olsen et al., 2021).

Therefore, although many studies have examined the effects of regular physical exercise on the cardiovascular and respiratory systems, the specific effects of physical exercise on the pathological mechanisms of pulmonary hypertension have not been fully studied, especially the lack of exercise and the mechanism of pulmonary artery vascular hyperplasia. Therefore, future research on aerobic exercise and vascular hyperplasia should be increased.

### Pulmonary artery hypertension and recommended exercise

**FIGURE 2:**
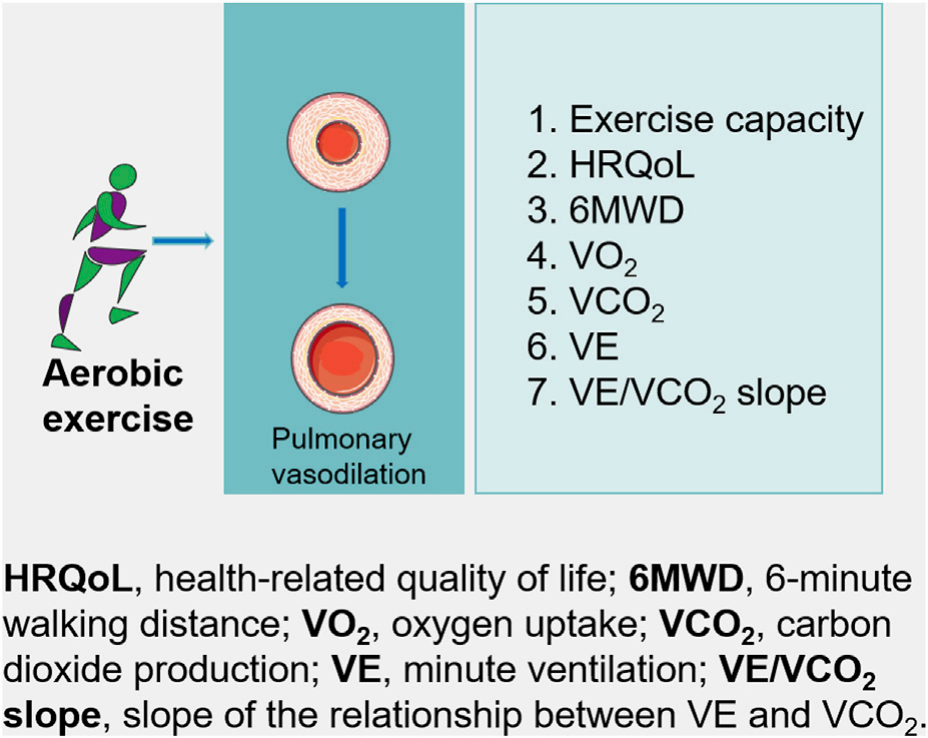
For patients with pulmonary hypertension, aerobic exercise may lead to substantial improvements in exercise capacity, which may improves pulmonary hypertension.

Patients with PH have reduced exercise capacity and quality of life. Despite initial concerns that exercise training might exacerbate symptoms in such patients, several studies have reported that exercise rehabilitation training improves function and quality of life (Figure 2).

European Respiratory Society published a statement on exercise training in PH reporting improvements in exercise capacity, muscular function, quality of life and potentially right ventricular function (Grünig et al., 2019). However, it was noted that further studies were needed to consolidate these findings and the impact of exercise training on disease risk profiles and to establish optimal training methodology. European Cardiac and Respiratory Societies Guidelines for the diagnosis and treatment of PH recommend supervised exercise training for people with PAH under medical therapy (Class of recommendation: 1; level of evidence A) (Humbert et al., 2022). With the broadening of international acceptance of exercise training in PH, albeit based on a limited body of evidence. In patients with PH, supervised exercise-based rehabilitation may result in a large increase in exercise capacity. Changes in exercise capacity remain heterogeneous and cannot be explained by subgroup analysis. It is likely that exercise-based rehabilitation increases health-related quality of life (HRQoL) and is probably not associated with an increased risk of a serious adverse events. Exercise training may result in a large reduction in mean pulmonary arterial pressure (Morris et al., 2023).

The beneficial effects of exercise rehabilitation training were mainly demonstrated by improvements in peak oxygen uptake (pVO_2_), 6-minute walk distance (6MWD), hemodynamics, cardiorespiratory fitness, and exercise capacity in patients with different types of PH.

PH patients receiving PH medication and inoperable patients were randomly assigned to training and control groups. The training regimen consisted of low-load intermittent cycle ergometer training, walking, low weight (500–1,000 g) dumbbell training for a single muscle group, and respiratory training for at least 1.5 h per day. Results showed a significant increase in pVO_2_/kg in the training group after 15 weeks (mean increase of 24.3% relative to baseline). The training group also showed significant improvements in cardiac index, mean pulmonary artery pressure, pulmonary vascular resistance, 6MWD, quality of life scores and exercise tolerance at rest and during exercise (Ehlken et al., 2016). It was also shown that 8 weeks of exercise significantly improved a predictor of death in PH: cardiorespiratory fitness (Santos-Lozano et al., 2019). The efficacy of low-dose exercise and breathing training as an add-on treatment for severe chronic PH. Their effects are a powerful complement to the beneficial results of new medical treatments.

Cardiopulmonary exercise testing (CPET) is a comprehensive methodology well studied in PAH with roles in diagnosis, treatment response, and prognosis. Submaximal and maximal exercise data is a valuable tool in detecting abnormal hemodynamics associated with exercise-induced and resting pulmonary hypertension as well as right ventricular dysfunction. Pulsatile pulmonary vascular pressure-flow relationships in PH allow for the assessment of RV hydraulic load. 12 weeks of aerobic training can significantly improve right ventricular systolic blood pressure in patients with PH (Atef and Abdeen, 2021). The increased granularity of CPET may help further risk stratify patients to inform prognosis and better individualize treatment decisions (Sherman and Saggar, 2023). Chronic obstructive pulmonary disease or diffuse interstitial lung disease, CPET helps to understand the nature of exertional limitation. Different studies have shown that the presence of PH significantly reduces peak oxygen consumption and oxygen pulse volume in these chronic lung diseases, confirming that the typical ventilatory limitation of the underlying respiratory disease is associated with cardiovascular restriction (Blanco et al., 2020).

Decreased exercise capacity is a common feature of PH. The 6-min walk test (6MWT) is often the most common method incorporated into the clinical assessment of the disease (Babu et al., 2017). The test can be used to assess the efficacy of interventions and to provide prognostic information (Holland et al., 2014). In the past, the PM6M was used to assess the efficacy of treatment for PH, and it is now used as one of the basic elements in the multiparametric assessment of risk of death (Boucly et al., 2014). For example, in PH due to chronic thromboembolic disease, which can be resolved surgically, PM6M is routinely performed before and after pulmonary endarterectomy as a tool to assess disease severity, functional capacity and prognosis (Richter et al., 2014).

In conclusion, for patients with PH, supervised exercise rehabilitation may lead to substantial improvements in exercise capacity, which in turn improves PH.
